# {2-[(2-Carbamothiol­ylhydrazin-1-yl­idene-κ^2^ *N* ^1^,*S*)meth­yl]-6-hy­droxy­phenolato-κ*O* ^1^}(triphenyl­phosphine-κ*P*)nickel(II) chloride

**DOI:** 10.1107/S1600536810031065

**Published:** 2010-08-11

**Authors:** Hana Bashir Shawish, Kong Wai Tan, M. Jamil Maah, Seik Weng Ng

**Affiliations:** a Department of Chemistry, University of Malaya, 50603 Kuala Lumpur, Malaysia

## Abstract

The deprotonated Schiff base ligand in the title compound, [Ni(C_8_H_8_N_3_O_2_S)(C_18_H_15_P)]Cl, functions as an *N*,*O*,*S*-chelating anion to the phosphine-coordinated Ni atom, which exists in a distorted square-planar geometry. The hy­droxy group forms an intra­molecular O—H⋯O hydrogen bond. The two amino groups of the cation are hydrogen-bond donors to the chloride anion; the hydrogen bonds generate a chain structure running along the *b* axis.

## Related literature

For the crystal structure of 2,3-dihy­droxy­benzaldehyde thio­semicarbazone hemihydrate, see: Swesi *et al.* (2006 ▶). For similar crystal structures containing a nickel(II) atom, see: García-Reynaldos *et al.* (2007 ▶).
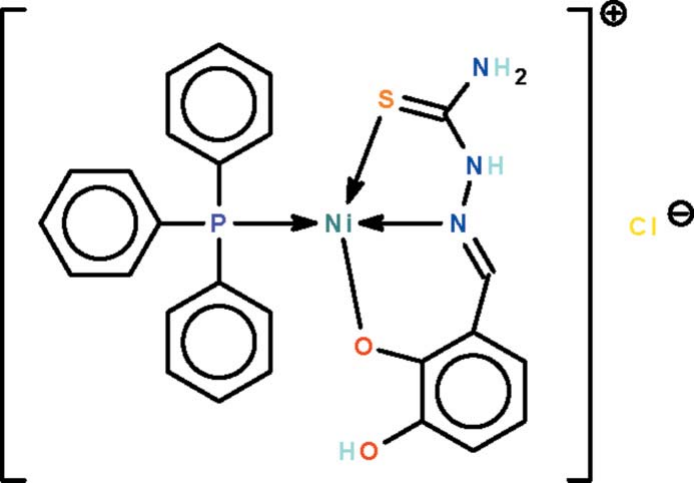


## Experimental

### 

#### Crystal data

[Ni(C_8_H_8_N_3_O_2_S)(C_18_H_15_P)]Cl
*M* *_r_* = 566.66
Orthorhombic, 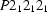

*a* = 7.7902 (4) Å
*b* = 14.6791 (7) Å
*c* = 21.7410 (11) Å
*V* = 2486.2 (2) Å^3^
*Z* = 4
Mo *K*α radiation
μ = 1.07 mm^−1^
*T* = 100 K
0.35 × 0.25 × 0.20 mm

#### Data collection

Bruker SMART APEX diffractometer
Absorption correction: multi-scan (*SADABS*; Sheldrick, 1996 ▶) *T*_min_ = 0.707, *T*_max_ = 0.815
24072 measured reflections
5703 independent reflections
5490 reflections with *I* > 2σ(*I*)
*R*_int_ = 0.028

#### Refinement

*R*[*F* ^2^ > 2σ(*F* ^2^)] = 0.021
*wR*(*F* ^2^) = 0.054
*S* = 1.02
5703 reflections
332 parameters
4 restraints
H atoms treated by a mixture of independent and constrained refinement
Δρ_max_ = 0.29 e Å^−3^
Δρ_min_ = −0.21 e Å^−3^
Absolute structure: Flack (1983 ▶), 2468 Friedel pairs
Flack parameter: −0.011 (7)

### 

Data collection: *APEX2* (Bruker, 2009 ▶); cell refinement: *SAINT* (Bruker, 2009 ▶); data reduction: *SAINT*; program(s) used to solve structure: *SHELXS97* (Sheldrick, 2008 ▶); program(s) used to refine structure: *SHELXL97* (Sheldrick, 2008 ▶); molecular graphics: *X-SEED* (Barbour, 2001 ▶); software used to prepare material for publication: *publCIF* (Westrip, 2010 ▶).

## Supplementary Material

Crystal structure: contains datablocks global, I. DOI: 10.1107/S1600536810031065/bt5315sup1.cif

Structure factors: contains datablocks I. DOI: 10.1107/S1600536810031065/bt5315Isup2.hkl

Additional supplementary materials:  crystallographic information; 3D view; checkCIF report

## Figures and Tables

**Table 1:** Selected bond lengths (Å)

Ni1—O1	1.847 (1)
Ni1—N1	1.897 (1)
Ni1—P1	2.1998 (4)
Ni1—S1	2.1416 (4)

**Table 2:** Hydrogen-bond geometry (Å, °)

*D*—H⋯*A*	*D*—H	H⋯*A*	*D*⋯*A*	*D*—H⋯*A*
O2—H1⋯O1	0.84 (1)	2.11 (3)	2.636 (2)	120 (2)
N2—H2⋯Cl1	0.86 (1)	2.17 (1)	3.016 (2)	167 (2)
N3—H3⋯Cl1^i^	0.85 (1)	2.46 (1)	3.275 (2)	161 (2)

Symmetry code: (i) 
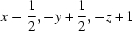
.

**Fig. 1.**
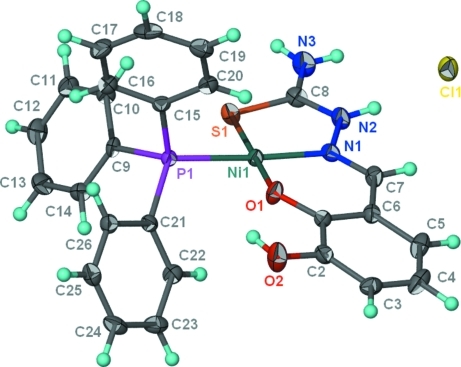
Anisotropic displacement ellipsoid plot (Barbour, 2001) of the title compound at the 70% probability level; hydrogen atoms are drawn as spheres of arbitrary radius.

**Fig. 2.**
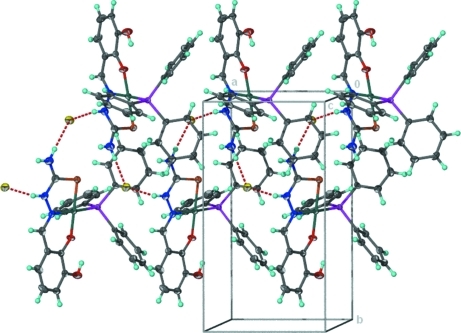
Hydrogen-bonded chain motif.

## supplementary crystallographic information

### Comment

Substituted 2-hydroxybenzaldehyde thiosemicarbazones are generally doubly-deprotonated in their nickel complexes, the dianion chelating to the metal atom through its nitrogen, oxygen and sulfur atoms. However, with the triphenylphosphine adducts of nickel 2-hydroxybenzaldehyde thiosemicarbonates, the Schiff base is only mono-deprotonated; the positive charge of the cation is balanced by a chloride counterion (García-Reynaldos *et al.*, 2007). The reaction of the 3-hydroxy substituted Schiff base with nickel chloride affords the analogous salt, 3-hydroxy-2-oxidobenzaldehydethiosemicarbazone)(triphenylphosphine)nickel(II) chloride (Scheme I). The coordination environment of nickel is a square plane made up of nitrogen, oxygen, phosphorus and sulfur atoms (Fig. 1). Adjacent ions are linked by N–H···Cl hydrogen bonds to generate a chain structure (Fig. 2).

### Experimental

2,3-Dihydroxybenzaldehyde thiosemicarbazone hemihydrate (Swesi *et al.*, 2006) (0.22 g, 1 mmol), triphenylphosphine (0.26, 1 mmol) and nickel chloride (0.13 g, 1 mmol) were heated in a methanol/ethanol (50 ml) for an hour. The brown solution was then set aside for the growth of crystals.

### Refinement

Carbon-bound H-atoms were placed in calculated positions (C—H 0.95 Å) and were included in the refinement in the riding model approximation, with *U*(H) set to 1.2*U*(C).

The amino and hydroxy H-atoms were located in a difference Fourier map, and were refined with distance restraints of N–H 0.86±0.01 and O–H 0.84±0.01 Å; their displacement paramters were freely refined.

### Crystal data

**Table:**

[Ni(C_8_H_8_N_3_O_2_S)(C_18_H_15_P)]Cl	*F*(000) = 1168
*M**_r_* = 566.66	*D*_x_ = 1.514 Mg m^−^^3^
Orthorhombic, *P*2_1_2_1_2_1_	Mo *K*α radiation, λ = 0.71073 Å
Hall symbol: P 2ac 2ab	Cell parameters from 9879 reflections
*a* = 7.7902 (4) Å	θ = 2.8–28.3°
*b* = 14.6791 (7) Å	µ = 1.07 mm^−^^1^
*c* = 21.7410 (11) Å	*T* = 100 K
*V* = 2486.2 (2) Å^3^	Block, brown
*Z* = 4	0.35 × 0.25 × 0.20 mm

### Data collection

**Table:**

Bruker SMART APEX diffractometer	5703 independent reflections
Radiation source: fine-focus sealed tube	5490 reflections with *I* > 2σ(*I*)
graphite	*R*_int_ = 0.028
ω scans	θ_max_ = 27.5°, θ_min_ = 1.7°
Absorption correction: multi-scan (*SADABS*; Sheldrick, 1996)	*h* = −10→10
*T*_min_ = 0.707, *T*_max_ = 0.815	*k* = −19→19
24072 measured reflections	*l* = −28→28

### Refinement

**Table:**

Refinement on *F*^2^	Secondary atom site location: difference Fourier map
Least-squares matrix: full	Hydrogen site location: inferred from neighbouring sites
*R*[*F*^2^ > 2σ(*F*^2^)] = 0.021	H atoms treated by a mixture of independent and constrained refinement
*wR*(*F*^2^) = 0.054	*w* = 1/[σ^2^(*F*_o_^2^) + (0.032*P*)^2^ + 0.4343*P*] where *P* = (*F*_o_^2^ + 2*F*_c_^2^)/3
*S* = 1.02	(Δ/σ)_max_ = 0.001
5703 reflections	Δρ_max_ = 0.29 e Å^−^^3^
332 parameters	Δρ_min_ = −0.21 e Å^−^^3^
4 restraints	Absolute structure: Flack (1983), 2468 Friedel pairs
Primary atom site location: structure-invariant direct methods	Flack parameter: −0.011 (7)

### Fractional atomic coordinates and isotropic or equivalent isotropic displacement parameters (Å^2^)

**Table:**

	*x*	*y*	*z*	*U*_iso_*/*U*_eq_	
Ni1	1.20955 (2)	0.505537 (13)	0.401439 (8)	0.01255 (5)	
Cl1	1.73655 (6)	0.39315 (3)	0.57199 (2)	0.02565 (10)	
S1	1.17398 (5)	0.36636 (3)	0.42809 (2)	0.01724 (9)	
P1	1.03089 (5)	0.49177 (3)	0.323635 (17)	0.01239 (8)	
O1	1.24123 (15)	0.62350 (7)	0.37422 (5)	0.0187 (2)	
O2	1.19946 (19)	0.78945 (8)	0.33035 (6)	0.0287 (3)	
N1	1.37566 (17)	0.51529 (9)	0.46493 (6)	0.0149 (3)	
N2	1.42733 (19)	0.43510 (10)	0.49349 (7)	0.0185 (3)	
N3	1.3866 (2)	0.28145 (11)	0.50486 (8)	0.0241 (3)	
C1	1.31436 (19)	0.68926 (10)	0.40627 (7)	0.0142 (3)	
C2	1.2891 (2)	0.77878 (11)	0.38354 (8)	0.0176 (3)	
C3	1.3508 (2)	0.85370 (11)	0.41499 (8)	0.0190 (3)	
H3A	1.3282	0.9134	0.4002	0.023*	
C4	1.4466 (3)	0.84135 (12)	0.46872 (8)	0.0240 (4)	
H4A	1.4888	0.8928	0.4904	0.029*	
C5	1.4800 (2)	0.75525 (13)	0.49031 (8)	0.0214 (4)	
H5	1.5483	0.7476	0.5261	0.026*	
C6	1.4135 (2)	0.67776 (11)	0.45966 (7)	0.0155 (3)	
C7	1.4512 (2)	0.58908 (11)	0.48329 (8)	0.0170 (3)	
H7	1.5366	0.5840	0.5143	0.020*	
C8	1.3423 (2)	0.35953 (11)	0.47936 (8)	0.0178 (3)	
C9	0.8816 (2)	0.39672 (11)	0.32651 (8)	0.0148 (3)	
C10	0.9418 (2)	0.30780 (11)	0.31890 (8)	0.0185 (3)	
H10	1.0577	0.2979	0.3071	0.022*	
C11	0.8346 (2)	0.23421 (12)	0.32831 (8)	0.0217 (4)	
H11	0.8771	0.1740	0.3234	0.026*	
C12	0.6650 (2)	0.24820 (12)	0.34488 (8)	0.0233 (4)	
H12	0.5919	0.1977	0.3525	0.028*	
C13	0.6022 (2)	0.33652 (13)	0.35029 (9)	0.0243 (4)	
H13	0.4846	0.3460	0.3599	0.029*	
C14	0.7092 (2)	0.41065 (11)	0.34182 (8)	0.0201 (3)	
H14	0.6658	0.4708	0.3464	0.024*	
C15	1.1516 (2)	0.47966 (10)	0.25280 (7)	0.0137 (3)	
C16	1.0725 (2)	0.44338 (11)	0.20070 (8)	0.0186 (3)	
H16	0.9612	0.4172	0.2037	0.022*	
C17	1.1566 (3)	0.44565 (12)	0.14459 (8)	0.0243 (4)	
H17	1.1017	0.4219	0.1090	0.029*	
C18	1.3205 (2)	0.48240 (12)	0.13991 (8)	0.0235 (4)	
H18	1.3763	0.4851	0.1011	0.028*	
C19	1.4021 (2)	0.51502 (12)	0.19194 (8)	0.0216 (3)	
H19	1.5158	0.5380	0.1891	0.026*	
C20	1.3182 (2)	0.51420 (11)	0.24836 (7)	0.0179 (3)	
H20	1.3742	0.5372	0.2839	0.021*	
C21	0.8940 (2)	0.59074 (10)	0.31157 (8)	0.0137 (3)	
C22	0.8430 (2)	0.64323 (11)	0.36199 (8)	0.0174 (3)	
H22	0.8851	0.6291	0.4019	0.021*	
C23	0.7311 (2)	0.71597 (12)	0.35396 (8)	0.0205 (4)	
H23	0.6977	0.7517	0.3884	0.025*	
C24	0.6678 (2)	0.73673 (11)	0.29596 (8)	0.0212 (4)	
H24	0.5907	0.7862	0.2907	0.025*	
C25	0.7181 (2)	0.68466 (11)	0.24565 (8)	0.0188 (3)	
H25	0.6748	0.6986	0.2059	0.023*	
C26	0.8313 (2)	0.61229 (11)	0.25321 (8)	0.0159 (3)	
H26	0.8661	0.5774	0.2186	0.019*	
H1	1.167 (4)	0.7369 (10)	0.3206 (13)	0.066 (9)*	
H2	1.5176 (19)	0.4328 (14)	0.5163 (8)	0.024 (5)*	
H3	1.345 (3)	0.2305 (10)	0.4935 (10)	0.034 (6)*	
H4	1.470 (2)	0.2785 (16)	0.5305 (9)	0.041 (7)*	

### Atomic displacement parameters (Å^2^)

**Table:**

	*U*^11^	*U*^22^	*U*^33^	*U*^12^	*U*^13^	*U*^23^
Ni1	0.01379 (9)	0.01142 (9)	0.01246 (9)	0.00043 (8)	−0.00276 (7)	0.00082 (8)
Cl1	0.0260 (2)	0.0220 (2)	0.0290 (2)	0.00243 (16)	−0.01141 (18)	−0.00098 (17)
S1	0.01566 (18)	0.01508 (18)	0.0210 (2)	−0.00126 (15)	−0.00263 (16)	0.00560 (15)
P1	0.01259 (17)	0.01158 (18)	0.01300 (18)	−0.00005 (16)	−0.00142 (13)	−0.00061 (15)
O1	0.0255 (6)	0.0114 (5)	0.0190 (6)	−0.0023 (5)	−0.0093 (5)	0.0008 (4)
O2	0.0330 (7)	0.0164 (6)	0.0368 (7)	−0.0018 (6)	−0.0196 (6)	0.0063 (5)
N1	0.0150 (6)	0.0171 (7)	0.0126 (6)	0.0033 (5)	−0.0011 (5)	0.0028 (5)
N2	0.0188 (7)	0.0198 (7)	0.0168 (7)	0.0026 (6)	−0.0063 (6)	0.0040 (6)
N3	0.0264 (9)	0.0198 (8)	0.0261 (8)	0.0011 (6)	−0.0061 (7)	0.0075 (7)
C1	0.0125 (7)	0.0153 (7)	0.0148 (7)	−0.0003 (6)	0.0006 (6)	−0.0014 (6)
C2	0.0137 (7)	0.0168 (7)	0.0223 (8)	−0.0001 (6)	−0.0007 (7)	−0.0003 (6)
C3	0.0189 (8)	0.0138 (7)	0.0242 (9)	−0.0015 (6)	0.0055 (7)	−0.0019 (6)
C4	0.0313 (10)	0.0225 (9)	0.0183 (9)	−0.0107 (8)	0.0045 (7)	−0.0054 (7)
C5	0.0259 (9)	0.0244 (8)	0.0140 (8)	−0.0078 (7)	−0.0005 (7)	−0.0015 (7)
C6	0.0142 (7)	0.0183 (8)	0.0139 (8)	−0.0018 (6)	0.0002 (6)	−0.0011 (6)
C7	0.0164 (8)	0.0239 (8)	0.0108 (7)	−0.0009 (6)	−0.0014 (6)	0.0004 (6)
C8	0.0182 (8)	0.0186 (8)	0.0166 (8)	0.0013 (6)	0.0009 (6)	0.0041 (6)
C9	0.0147 (7)	0.0150 (7)	0.0146 (8)	−0.0040 (6)	−0.0027 (6)	−0.0019 (6)
C10	0.0165 (8)	0.0180 (8)	0.0209 (9)	0.0008 (7)	−0.0012 (7)	−0.0018 (6)
C11	0.0238 (9)	0.0143 (8)	0.0268 (9)	−0.0003 (7)	−0.0037 (8)	−0.0026 (7)
C12	0.0225 (9)	0.0226 (9)	0.0249 (9)	−0.0088 (7)	−0.0005 (7)	0.0014 (7)
C13	0.0149 (8)	0.0270 (9)	0.0312 (10)	−0.0025 (7)	0.0042 (7)	−0.0028 (8)
C14	0.0184 (8)	0.0180 (8)	0.0239 (9)	0.0020 (7)	0.0002 (7)	−0.0032 (6)
C15	0.0156 (7)	0.0109 (7)	0.0147 (7)	0.0024 (5)	−0.0006 (6)	−0.0004 (5)
C16	0.0182 (8)	0.0184 (8)	0.0191 (8)	0.0007 (6)	−0.0012 (7)	−0.0032 (6)
C17	0.0301 (10)	0.0252 (9)	0.0175 (8)	0.0055 (8)	−0.0033 (7)	−0.0079 (7)
C18	0.0286 (9)	0.0234 (9)	0.0187 (8)	0.0102 (7)	0.0080 (7)	−0.0007 (6)
C19	0.0176 (8)	0.0186 (8)	0.0286 (9)	0.0015 (7)	0.0051 (7)	0.0007 (7)
C20	0.0180 (8)	0.0157 (7)	0.0200 (7)	0.0011 (6)	−0.0008 (6)	−0.0024 (6)
C21	0.0117 (7)	0.0115 (7)	0.0178 (8)	−0.0005 (6)	−0.0003 (6)	0.0005 (6)
C22	0.0187 (8)	0.0165 (8)	0.0170 (8)	−0.0006 (6)	0.0004 (6)	−0.0002 (6)
C23	0.0200 (9)	0.0176 (7)	0.0237 (9)	0.0017 (7)	0.0073 (7)	−0.0015 (7)
C24	0.0147 (8)	0.0142 (7)	0.0346 (10)	0.0025 (6)	0.0032 (7)	0.0041 (7)
C25	0.0157 (8)	0.0179 (7)	0.0228 (8)	−0.0020 (7)	−0.0031 (7)	0.0041 (6)
C26	0.0156 (7)	0.0154 (7)	0.0169 (7)	−0.0007 (6)	−0.0019 (6)	−0.0001 (6)

### Geometric parameters (Å, °)

**Table:**

Ni1—O1	1.847 (1)	C10—C11	1.381 (2)
Ni1—N1	1.897 (1)	C10—H10	0.9500
Ni1—P1	2.1998 (4)	C11—C12	1.384 (3)
Ni1—S1	2.1416 (4)	C11—H11	0.9500
S1—C8	1.7240 (17)	C12—C13	1.391 (3)
P1—C15	1.8134 (16)	C12—H12	0.9500
P1—C9	1.8176 (17)	C13—C14	1.383 (2)
P1—C21	1.8211 (16)	C13—H13	0.9500
O1—C1	1.3198 (18)	C14—H14	0.9500
O2—C2	1.360 (2)	C15—C16	1.395 (2)
O2—H1	0.839 (10)	C15—C20	1.396 (2)
N1—C7	1.296 (2)	C16—C17	1.385 (2)
N1—N2	1.3904 (19)	C16—H16	0.9500
N2—C8	1.328 (2)	C17—C18	1.390 (3)
N2—H2	0.861 (9)	C17—H17	0.9500
N3—C8	1.319 (2)	C18—C19	1.383 (3)
N3—H3	0.850 (10)	C18—H18	0.9500
N3—H4	0.857 (10)	C19—C20	1.390 (2)
C1—C6	1.405 (2)	C19—H19	0.9500
C1—C2	1.418 (2)	C20—H20	0.9500
C2—C3	1.381 (2)	C21—C26	1.396 (2)
C3—C4	1.398 (2)	C21—C22	1.397 (2)
C3—H3A	0.9500	C22—C23	1.390 (2)
C4—C5	1.373 (3)	C22—H22	0.9500
C4—H4A	0.9500	C23—C24	1.388 (3)
C5—C6	1.416 (2)	C23—H23	0.9500
C5—H5	0.9500	C24—C25	1.391 (2)
C6—C7	1.430 (2)	C24—H24	0.9500
C7—H7	0.9500	C25—C26	1.391 (2)
C9—C10	1.397 (2)	C25—H25	0.9500
C9—C14	1.399 (2)	C26—H26	0.9500
			
O1—Ni1—N1	94.09 (5)	C11—C10—C9	120.68 (16)
O1—Ni1—S1	176.98 (4)	C11—C10—H10	119.7
N1—Ni1—S1	87.90 (4)	C9—C10—H10	119.7
O1—Ni1—P1	85.66 (4)	C10—C11—C12	119.98 (16)
N1—Ni1—P1	176.19 (4)	C10—C11—H11	120.0
S1—Ni1—P1	92.207 (17)	C12—C11—H11	120.0
C8—S1—Ni1	97.58 (6)	C11—C12—C13	119.74 (17)
C15—P1—C9	106.62 (7)	C11—C12—H12	120.1
C15—P1—C21	105.05 (7)	C13—C12—H12	120.1
C9—P1—C21	104.04 (7)	C14—C13—C12	120.68 (17)
C15—P1—Ni1	109.50 (5)	C14—C13—H13	119.7
C9—P1—Ni1	116.68 (5)	C12—C13—H13	119.7
C21—P1—Ni1	114.07 (5)	C13—C14—C9	119.68 (16)
C1—O1—Ni1	125.06 (10)	C13—C14—H14	120.2
C2—O2—H1	105 (2)	C9—C14—H14	120.2
C7—N1—N2	116.02 (13)	C16—C15—C20	119.55 (15)
C7—N1—Ni1	126.64 (12)	C16—C15—P1	119.83 (12)
N2—N1—Ni1	117.29 (10)	C20—C15—P1	120.34 (12)
C8—N2—N1	117.35 (13)	C17—C16—C15	119.78 (16)
C8—N2—H2	120.6 (14)	C17—C16—H16	120.1
N1—N2—H2	121.7 (14)	C15—C16—H16	120.1
C8—N3—H3	122.9 (16)	C16—C17—C18	120.56 (16)
C8—N3—H4	121.0 (17)	C16—C17—H17	119.7
H3—N3—H4	115 (2)	C18—C17—H17	119.7
O1—C1—C6	125.86 (14)	C19—C18—C17	119.80 (16)
O1—C1—C2	115.72 (14)	C19—C18—H18	120.1
C6—C1—C2	118.42 (14)	C17—C18—H18	120.1
O2—C2—C3	120.53 (15)	C18—C19—C20	120.15 (16)
O2—C2—C1	118.33 (14)	C18—C19—H19	119.9
C3—C2—C1	121.13 (15)	C20—C19—H19	119.9
C2—C3—C4	119.75 (16)	C19—C20—C15	120.09 (15)
C2—C3—H3A	120.1	C19—C20—H20	120.0
C4—C3—H3A	120.1	C15—C20—H20	120.0
C5—C4—C3	120.41 (16)	C26—C21—C22	119.25 (15)
C5—C4—H4A	119.8	C26—C21—P1	121.10 (12)
C3—C4—H4A	119.8	C22—C21—P1	119.55 (13)
C4—C5—C6	120.62 (16)	C23—C22—C21	120.22 (15)
C4—C5—H5	119.7	C23—C22—H22	119.9
C6—C5—H5	119.7	C21—C22—H22	119.9
C1—C6—C5	119.56 (15)	C24—C23—C22	120.38 (16)
C1—C6—C7	121.27 (15)	C24—C23—H23	119.8
C5—C6—C7	119.17 (15)	C22—C23—H23	119.8
N1—C7—C6	123.88 (15)	C23—C24—C25	119.62 (15)
N1—C7—H7	118.1	C23—C24—H24	120.2
C6—C7—H7	118.1	C25—C24—H24	120.2
N3—C8—N2	119.87 (16)	C24—C25—C26	120.36 (16)
N3—C8—S1	121.41 (14)	C24—C25—H25	119.8
N2—C8—S1	118.71 (12)	C26—C25—H25	119.8
C10—C9—C14	119.17 (16)	C25—C26—C21	120.16 (16)
C10—C9—P1	119.88 (13)	C25—C26—H26	119.9
C14—C9—P1	120.69 (13)	C21—C26—H26	119.9
			
N1—Ni1—S1—C8	−8.42 (7)	C21—P1—C9—C10	162.94 (14)
P1—Ni1—S1—C8	167.76 (6)	Ni1—P1—C9—C10	−70.45 (15)
O1—Ni1—P1—C15	76.20 (7)	C15—P1—C9—C14	−133.63 (14)
S1—Ni1—P1—C15	−101.67 (5)	C21—P1—C9—C14	−22.91 (16)
O1—Ni1—P1—C9	−162.64 (7)	Ni1—P1—C9—C14	103.70 (14)
S1—Ni1—P1—C9	19.49 (6)	C14—C9—C10—C11	−2.2 (3)
O1—Ni1—P1—C21	−41.18 (7)	P1—C9—C10—C11	172.08 (13)
S1—Ni1—P1—C21	140.95 (6)	C9—C10—C11—C12	0.6 (3)
N1—Ni1—O1—C1	−18.45 (13)	C10—C11—C12—C13	1.8 (3)
P1—Ni1—O1—C1	165.37 (13)	C11—C12—C13—C14	−2.7 (3)
O1—Ni1—N1—C7	9.88 (14)	C12—C13—C14—C9	1.2 (3)
S1—Ni1—N1—C7	−172.40 (14)	C10—C9—C14—C13	1.2 (3)
O1—Ni1—N1—N2	−167.58 (11)	P1—C9—C14—C13	−172.98 (14)
S1—Ni1—N1—N2	10.15 (11)	C9—P1—C15—C16	33.87 (15)
C7—N1—N2—C8	174.49 (15)	C21—P1—C15—C16	−76.15 (14)
Ni1—N1—N2—C8	−7.78 (19)	Ni1—P1—C15—C16	160.94 (11)
Ni1—O1—C1—C6	15.1 (2)	C9—P1—C15—C20	−152.17 (12)
Ni1—O1—C1—C2	−165.23 (11)	C21—P1—C15—C20	97.81 (13)
O1—C1—C2—O2	−2.6 (2)	Ni1—P1—C15—C20	−25.10 (13)
C6—C1—C2—O2	177.13 (15)	C20—C15—C16—C17	−2.9 (2)
O1—C1—C2—C3	176.23 (15)	P1—C15—C16—C17	171.13 (13)
C6—C1—C2—C3	−4.0 (2)	C15—C16—C17—C18	1.1 (3)
O2—C2—C3—C4	−178.30 (16)	C16—C17—C18—C19	1.6 (3)
C1—C2—C3—C4	2.9 (3)	C17—C18—C19—C20	−2.4 (3)
C2—C3—C4—C5	0.2 (3)	C18—C19—C20—C15	0.6 (2)
C3—C4—C5—C6	−2.0 (3)	C16—C15—C20—C19	2.0 (2)
O1—C1—C6—C5	−178.13 (16)	P1—C15—C20—C19	−171.93 (12)
C2—C1—C6—C5	2.2 (2)	C15—P1—C21—C26	31.84 (15)
O1—C1—C6—C7	2.8 (3)	C9—P1—C21—C26	−80.02 (14)
C2—C1—C6—C7	−176.86 (16)	Ni1—P1—C21—C26	151.76 (12)
C4—C5—C6—C1	0.8 (3)	C15—P1—C21—C22	−151.69 (13)
C4—C5—C6—C7	179.84 (17)	C9—P1—C21—C22	96.45 (14)
N2—N1—C7—C6	−179.36 (15)	Ni1—P1—C21—C22	−31.77 (14)
Ni1—N1—C7—C6	3.2 (2)	C26—C21—C22—C23	0.1 (2)
C1—C6—C7—N1	−12.3 (3)	P1—C21—C22—C23	−176.48 (13)
C5—C6—C7—N1	168.62 (16)	C21—C22—C23—C24	0.5 (3)
N1—N2—C8—N3	179.62 (15)	C22—C23—C24—C25	−0.5 (3)
N1—N2—C8—S1	−0.9 (2)	C23—C24—C25—C26	−0.2 (3)
Ni1—S1—C8—N3	−173.17 (14)	C24—C25—C26—C21	0.7 (2)
Ni1—S1—C8—N2	7.37 (14)	C22—C21—C26—C25	−0.7 (2)
C15—P1—C9—C10	52.22 (15)	P1—C21—C26—C25	175.82 (12)

### Hydrogen-bond geometry (Å, °)

**Table:**

*D*—H···*A*	*D*—H	H···*A*	*D*···*A*	*D*—H···*A*
O2—H1···O1	0.84 (1)	2.11 (3)	2.636 (2)	120 (2)
N2—H2···Cl1	0.86 (1)	2.17 (1)	3.016 (2)	167 (2)
N3—H3···Cl1^i^	0.85 (1)	2.46 (1)	3.275 (2)	161 (2)

Symmetry codes: (i) *x*−1/2, −*y*+1/2, −*z*+1.

## References

[bb1] Barbour, L. J. (2001). *J. Supramol. Chem.* **1**, 189–191.

[bb2] Bruker (2009). *APEX2* and *SAINT* Bruker AXS Inc., Madison, Wisconsin, USA.

[bb3] Flack, H. D. (1983). *Acta Cryst.* A**39**, 876–881.

[bb4] García-Reynaldos, P. X., Hernández-Ortega, S., Toscano, R. A. & Valdés-Martínez, J. (2007). *Supramol. Chem.* **19**, 613–619.

[bb5] Sheldrick, G. M. (1996). *SADABS* University of Göttingen, Germany.

[bb6] Sheldrick, G. M. (2008). *Acta Cryst.* A**64**, 112–122. 10.1107/S0108767307043930 18156677

[bb7] Swesi, A. T., Farina, Y., Kassim, M. & Ng, S. W. (2006). *Acta Cryst.* E**62**, o5457–o5458.

[bb8] Westrip, S. P. (2010). *J. Appl. Cryst.* **43**, 920–925.

